# Thermal Properties of Porous Mullite Ceramics Modified with Microsized ZrO_2_ and WO_3_

**DOI:** 10.3390/ma15227935

**Published:** 2022-11-10

**Authors:** Ludmila Mahnicka-Goremikina, Ruta Svinka, Visvaldis Svinka, Liga Grase, Inna Juhnevica, Maris Rundans, Vadims Goremikins, Sanat Tolendiuly, Sergey Fomenko

**Affiliations:** 1 Institute of Materials and Surface Engineering, Faculty of Materials Science and Applied Chemistry, Riga Technical University, Paula Valdena St. 3/7, LV-1048 Riga, Latvia; 2 Institute of Structural Engineering and Reconstruction, Riga Technical University, Kipsalas St. 6A, LV-1048 Riga, Latvia; 3 Space Engineering Department, AUPET Named G. Daukeev, Baitursynov St., 126/1, Almaty 050013, Kazakhstan; 4 Institute of Combustion Problems, Bogenbay Batyr St. 172, Almaty 050012, Kazakhstan

**Keywords:** mullite, porous ceramic, zirconia, tungsten oxide, thermal conductivity, thermal shock

## Abstract

Mullite ceramics are well known as materials with a high temperature stability, strength and creep resistance. In this research, the effect of a modification with magnesia-stabilized zirconia and yttria-stabilized zirconia, separately, as well as in a mixture with WO_3_, in 1:1 and 1:2 ratios on the thermal properties of porous mullite ceramics was investigated. The porous mullite-containing ceramics were prepared by a slip casting of the concentrated slurry of raw materials with the addition of a suspension of Al paste for the pore formation due to the H_2_ evolution as a result of the reaction of Al with water. The formed samples were sintered at 1600 °C and the holding time was 1 h. The materials were characterized using X-ray diffractometry, scanning electron microscopy, mercury porosimetry, the laser flash contactless method, thermal shock resistance testing and the non-destructive impulse excitation method for determining the elasticity modulus. The modification of the porous mullite ceramic with a mixture of ZrO_2_ and WO_3_ oxides had a positive effect by decreasing the thermal conductivity, due to the increased porosity, in comparison to the undoped samples and samples with only ZrO_2_. The doubling of the WO_3_ amount in the modifying oxide mixtures improved the ceramic thermal shock resistance. The porous mullite ceramics which were modified with magnesia-stabilized zirconia (2.8 mol% MgO) and WO_3_ had a lower thermal conductivity and improved thermal shock resistance than the samples with yttria-stabilized zirconia (8 mol% Y_2_O_3_) and WO_3_.

## 1. Introduction

Special materials are needed to save thermal energy in the working space of thermal high-temperature units and prevent it from flowing into the environment. Such materials are called high-temperature thermal insulation materials. Thermal energy losses during high-temperature processes often exceed its theoretical need by several times. Climate-neutral manufacturing aims to boost the efficient use of fossil and energy resources by reducing air and water pollution, reducing heat losses and slowing down climate change. The investigation and use of porous high-temperature ceramics for thermal insulation help to achieve the goals of climate-neutral manufacturing and decrease the environmental degradation [1,2,3,4].

Ceramic materials such as corundum, cordierite, zirconia, mullite and multiphase composite ceramics are used for high-temperature engineering applications. In order to apply these ceramic materials, it is important to take into account their thermal properties such as their thermal conductivity, specific heat capacity, thermal expansion coefficient, resistance to sudden changes in temperature, thermal shock cracking and thermal shock induced fracture, as well as their mechanical properties [5,6,7,8,9,10,11,12,13].

Several layers of ceramic refractory material with different thermal resistances and thermal conductivities or ceramic composite materials with gradient properties are applied often in attempt to meet the aim of limiting the heat loss of certain equipment. For example, the cement kiln body can be divided into three layers, the working layer, the thermal-preservation layer and the thermal-insulation layer [6,7,14,15]. Layers can be made from different ceramic materials. Each layer has different thermal properties and its own role. The main properties of the working layer are its high thermal shock resistance, low thermal conductivity, high strength and long lifetime. The thermal-preservation layer must also have a high strength and lower thermal conductivity. The thermal-insulation layer has an ultra-low thermal conductivity to prevent heat loss and ensure the thermal protection of the other layers. The same situation is encountered in the thermal insulation of a spacecraft, where the thermal insulation consists of several layers [8,9,10,11,12,14]. Chen al. investigated the specific facing from composite material–multilayer mullite-based brick and porous plates, in which the thermal properties change along a gradient [6]. It is difficult to choose layers of materials or form a composite material such that the materials’ components are physically and chemically compatible with one another. In addition, layering increases the load on the construction. It is important that there is no peeling or destruction during the operation under elevated temperature conditions [6,7,8,9]. In the case where thermal-insulating ceramics are used at high temperatures or extreme conditions, such as rapid increase and decrease in temperature, the introduced thermal shock may produce microcracks, whose growth and development can cause the structural failure of the component [10,11].

In terms of the economy and technology, it is beneficial when the insulation part of the thermal technical equipment and spaceships consists of one type of material with a low bulk density that combines a lot of functions and properties. There are difficulties in searching for ceramic candidates for thermal insulators. The first is the selection of a ceramic material oxide or composition of oxides that will have a low thermal conductivity. The second difficulty is achieving a low bulk density due to the certain porosity. The presence of pores with an effective pore size leads to a significant reduction in the thermal conductivity compared to a dense material. The third difficulty is obtaining ceramic materials that will simultaneously have low parameters, such as linear thermal expansion coefficients and thermal conductivity coefficients [13,16]. The use of multi-phase polycrystalline ceramics can solve this problem. It is also important to take into account the specific heat capacity of heat insulation ceramics. The specific heat capacity is defined as the quantity of heat absorbed per unit mass of the material when its temperature increases by 1 K [17]. More heat energy is required to increase the temperature of materials with a high specific heat capacity than ones with a low specific heat capacity. Depending on the purpose of the ceramics, the high or low specific heat capacity and thermal diffusivity of the ceramics must be taken into account [17,18,19].

Porous mullite-containing refractory ceramics are good candidates for a high-temperature thermal insulation and for reducing heat losses. Porous mullite-containing ceramics have a significant share in the field of technical ceramics for industrial and space fields [1,2,5]. The catalyst supports, filters for hot gases and molten metals, parts of burners, heat insulators of industrial furnaces, technical equipment and spaceships are produced from this type of ceramic. The choice of a refractory material for an application will be determined by the type of required functionality of the furnace, heating unit or refractory insulator component and the prevailing conditions, e.g., the gaseous atmosphere, the presence of slags and the type of metal charge [1,15,16,20,21,22].

In order to improve the mullite-containing ceramics, researchers investigated the influence of modifying such ceramics with different oxides on the thermal properties. Xu et al. sintered a cordierite–mullite–corundum composite with added Sm_2_O_3_ and achieved an increase in the thermal shock resistance and a decrease in the thermal conductivity (6.81 W/mK) and thermal expansion (5.96 × 10^−6^ °C^−1^) [23]. Li et al. synthesized columnar self-reinforced mullite porous ceramics by adding V_2_O_5_ at 1350–1550 °C and achieved a porosity of about 63% and a thermal conductivity of about 1.04 W/mK [24]. The use of Ho_2_O_3_ [25], Gd_2_O_3_ [26] and HfO_2_ [27] improved the thermal shock resistance and thermal durability of mullite-containing ceramics. There is still ongoing research related to the investigation of and improvement in the thermo-mechanical properties of mullite and alumina–mullite ceramic composites with zirconia or zirconia–zircon components [28,29,30,31,32,33]. The thermal conductivity, thermal diffusivity and specific heat capacity of ceramic samples could be obtained using different international test standards, provided in Table 1.

The application of WO_3_ as an additive or raw component for the formation of an additional crystalline phase is relevant in different fields such as gas sensing, chromogenic, photocatalytic and emerging applications (biomedical, antibiotic and artificial intelligence) [46]. The use of WO_3_ for a ceramic modification has not been extensively investigated. The main aim of the research work was the formation of the thermal-insulating mullite ceramic material with a high porosity, a high thermal shock resistance, at the same time as low linear thermal expansion coefficients, low thermal conductivity coefficients and a low specific heat capacity. The main tasks of the investigation were the modification of porous mullite ceramic with ZrO_2_ and WO_3_ oxides and the analysis of the thermal properties depending on the chemical compositions, structural features, porosity and pore morphology. Such ceramic materials will help reduce the heat loss and withstand the rapid temperature fluctuations [9,12,13,14,15,16].

## 2. Materials and Methods

### 2.1. Materials

Two types of aluminas, α-Al_2_O_3_ (d_50_ = 2 μm) and γ-Al_2_O_3_ (d_50_ = 80 μm), were purchased from Nabalox, Nabaltec AG, Schwandorf, Germany. Kaolin (d_50_ = 1.5 μm; SiO_2_—56.5 wt.%, Al_2_O_3_—31.0 wt.%) was purchased from MEKA, Amberger Kaolinwerke, Hirschau, Germany. The magnesia-stabilized zirconia (2.8 mol% MgO) with d_50_ = 0.8 μm was obtained from Goodfellow, Huntingdon, UK. The yttria-stabilized zirconia (8 mol% Y_2_O_3_) with d_50_ = 0.5 μm, SiO_2_ amorphous with d_50_ = 3–5 μm and WO_3_ with d_50_ = 5 μm were acquired from GetNanoMaterials, Saint-Cannat, France. Aluminum paste (solid content of 70 ± 2%) with d_50_ = 12 μm was purchased from Aquapor-9008, Schlenk Metallic Pigments GmbH, Roth, Germany.

### 2.2. Material Proportions

The base of the mullite ceramic was prepared from two types of aluminas (α-Al_2_O_3_ and γ-Al_2_O_3_), amorphous SiO_2_ and kaolin. The ratio of Al_2_O_3_ to SiO_2_ was 2.57:1 due to the mullite stoichiometric composition. The quantity of γ-Al_2_O_3_ was three times more than the quantity of α-Al_2_O_3_. Kaolin was used at 30 wt.%. Oxides such as yttria-stabilized zirconia (8 mol% Y_2_O_3_), magnesia-stabilized zirconia (2.8 mol% MgO) and WO_3_ were used for the ceramic modifications. Both oxides were also used together in a mixture for modifying the mullite ceramics. The ratios of the different stabilized zirconia and tungsten oxide were 1:1 and 1:2.

### 2.3. Sample Preparation Methods

A slip casting of the concentrated slurry of raw materials was used for the sample preparation. The water content of the concentrated slurry was 38–40 wt.%. First, the dry raw materials were mixed in a dry state. Then, its suspension was created with distilled water and mixed for 10 min with a mechanical mixer to obtain a uniform particle distribution in the slurry. The suspension of aluminum paste was added into the raw material suspension and mixed for about 7–10 min. Then, the raw material slurry was slip casted into the mold. The porosity of the mullite ceramics was obtained due to the hydrogen gas evolution as a result of the reaction between the aluminum paste and water. The formed initial pores became visually noticeable 15–20 min after the slip casting. The pore formation took 1 to 3 h. After that, the samples were dried for 24 h at 20–25 °C and then for 24 h at 100 °C. The dried samples were sintered at 1600 °C with a 250 °C/h (4.2 °C/min) heating rate, and the holding time at the maximum temperature was 1 h. The cooling process of the fired samples was as slow as the heating process.

### 2.4. XRD Analysis

The phase compositions of the sintered materials were characterized by X-ray diffraction analysis (XRD; Rigaku Ultima + (Japan)) with CuK_α_ radiation, a voltage on the Cu anode of 30 kV, a current intensity of 20 mA, a range of the measurement angle of 5–60 2*θ*° and a speed of the goniometer of 2°/min).

### 2.5. SEM Analysis

The morphology of the prepared samples was observed by using scanning electron microscopes: a TableTop SEM Hitachi TM3000 (Japan) at an electron beam energy of 5 keV and 15 keV, and a high-resolution SEM FEI Nova NanoSEM 650 (the Netherlands) at an electron beam energy of 10 keV. Metal coating sputtering was not used because the structures were observed in the low vacuum mode.

### 2.6. Apparent Porosity

The apparent porosity was mathematically calculated and based on the Archimedes’ principle (European standard EN 623-2) after soaking the samples in distilled water. The apparent porosity *P* is the ratio of the total volume of the open pores in a porous body to its bulk volume. The apparent porosity *P* was calculated as a percentage using the following Equation (1):

*P* = ((m_3_ − m_1_)/(m_3_ − m_2_)) × 100, (1)

where m_1_ is the mass of the dry test piece in grams, m_2_ is the apparent mass of the immersed test piece in grams and m_3_ is the mass of the soaked test piece in grams.

### 2.7. Hg Porosimetry

The pore size distribution of the porous mullite ceramic was analyzed by a mercury porosimeter (Quantachrome, Pore Master 33, USA). Mercury intrusion porosimetry makes it possible to obtain data on the pores in a material in a limited range from 0.006 μm to 1000 μm. 

### 2.8. Thermal Analysis

The specific heat capacity and thermal conductivity measurements and the calculation of the thermal diffusivity of the samples were carried out using the laser flash contactless method using the universal equipment table-top instrument Netzsch LFA 457 MicroFlash, Germany. The measurements were carried out in the temperature range from 25 °C to 1100 °C. The sample dimensions were 10 mm × 10 mm and the thickness was 3 mm.

Thermal diffusivity (α with the unit mm^2^/s) is a material-specific property for characterizing an unsteady heat conduction. This value describes how fast heat diffuses through the material. The thermal diffusivity was calculated from the following Equation (2):

α = κ/(ρCp),(2)

where κ is the thermal conductivity (W/(m·K)), ρ is the density (kg/m^3^) and Cp is the specific heat capacity (J/kg/K) [18,19].

### 2.9. Thermal Shock Resistance Testing

The thermal shock resistance of the ceramics was determined during 10 cycles of thermal shock corresponding to the scheme 20 °C → 1000 °C → 20 °C with an exposure for 1 h at 1000 °C.

### 2.10. Elasticity Modulus Determination

The change in the elasticity modulus was measured before and after the 1st, 2nd, 5th and 10th thermal shock test by a non-destructive impulse excitation method (equipment Buzz-O-Sonic 5.0; BuzzMac International, LLC, USA). The non-destructive method provides an opportunity to examine the same sample after each thermal shock cycle, which allowed for more precise results.

## 3. Results and Discussion

### 3.1. Mineralogical Phase Composition

**Figure 1:**
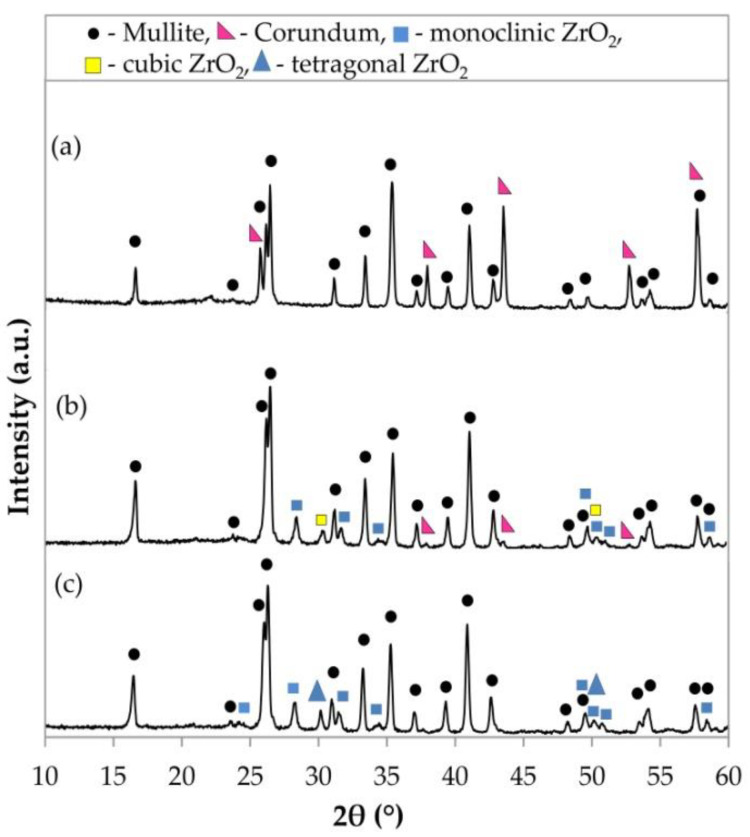
X-ray diffraction data of the sintered porous ceramic materials: (**a**) undoped samples, (**b**) samples with YSZ and (**c**) samples with MSZ.

**Figure 2:**
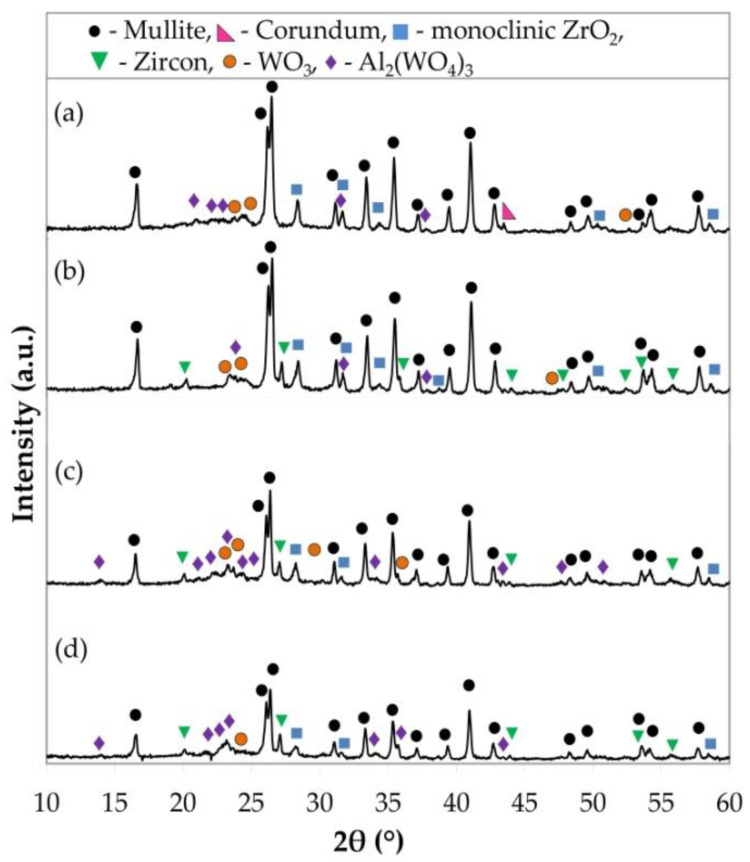
X-ray diffraction data of the sintered porous ceramic materials: (**a**) with YSZ:WO_3_ (1:1), (**b**) with MSZ:WO_3_ (1:1), (**c**) with YSZ:WO_3_ (1:2) and (**d**) with MSZ:WO_3_ (1:2).

The XRD patterns of the prepared materials are shown in Figure 1 and Figure 2. Comparing the experimental diffraction pattern with those from the International Centre of Diffraction Data (ICDD), the crystalline phases of the undoped samples correspond to mullite and corundum (Figure 1a).

The samples with yttria-stabilized zirconia and magnesia-stabilized zirconia contained mullite and monoclinic and tetragonal ZrO_2_ (Figure 1b,c). The corundum phase remained in the samples with YSZ. The XRD patterns (Figure 2a,b) of the samples with a mixture of YSZ or MSZ and WO_3_ in a 1:1 ratio show the presence of mullite, monoclinic ZrO_2_, WO_3_ and aluminum tungstate in both the materials of such compositions. Zircon was formed additionally in samples with MSZ and WO_3_ (1:1). 

The samples with a mixture of YSZ or MSZ and WO_3_ in a 1:2 ratio have the main phase, the mullite phase (Figure 2c,d), as well as such phases as monoclinic ZrO_2_, zircon and aluminum tungstate. The presence of WO_3_ in the compositions with MSZ and WO_3_ (1:2) was less pronounced than in the samples with YSZ and WO_3_ (1:2). A detailed description of the crystalline phase formation was considered in a previous investigation, which was described in the article [47].

### 3.2. Macrostructure

**Figure 3:**
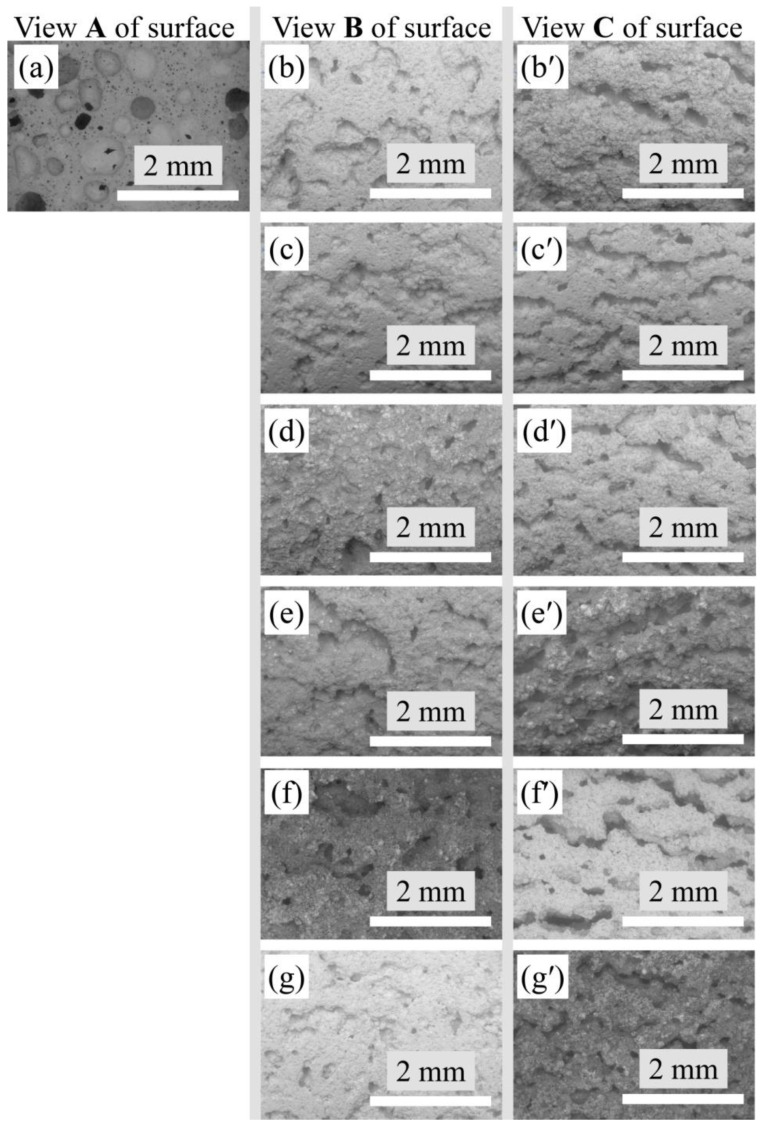
TableTop SEM micrographs illustrating the macrostructure of the investigated samples, magnification ×20: (**a**) view A of the undoped sample surface; (**b**–**g**) view B of the modified sample surface perpendicular to the base of the molds; (**b′**–**g′**) view C of the modified sample surface parallel to the base of the molds; (**b**,**b′**) samples with YSZ; (**c**,**c′**) with MSZ; (**d**,**d′**) with YSZ:WO_3_ (1:1); (**e**,**e′**) with MSZ:WO_3_ (1:1); (**f**,**f′**) with YSZ:WO_3_ (1:2); and (**g**,**g′**) with MSZ:WO_3_ (1:2).

The images of the porous mullite ceramic surface were obtained using a Table Top SEM and ×20 magnification (Figure 3). The pores have a spherical shape and approximately the same cross section as in the samples without the addition of modifying oxides (Figure 3a). 

The pores of the modified samples only with YSZ or MSZ as well as with these oxides and WO_3_ mixtures do not have the strong spherical form with a uniform diameter. All samples with modifying oxide additives are characterized by an elongated pore shape in the ceramic structure. It is important to note that these elongated pores are characterized by an expressed orientation in the ceramic structure. Such elongation and orientation occurred in the direction parallel to the base of the molds, respectively, parallel to the horizontal plane of the samples or along the sample length. When analyzing the photo of the surface of the modified samples along the pores and across the pores, it can be seen that to a greater extent, the pores are isolated from each other. The pores have to slit shapes with a partial networking because they have a joint intersection and branching, as well as a stomach and dead ends (Figure 3b–g). The length of the pores is 2–3 times greater than their width and height. The walls of the pores are not smooth but uneven due to the fact that large pores are formed from the merging of several smaller pores.

### 3.3. Microstructure

**Figure 4:**
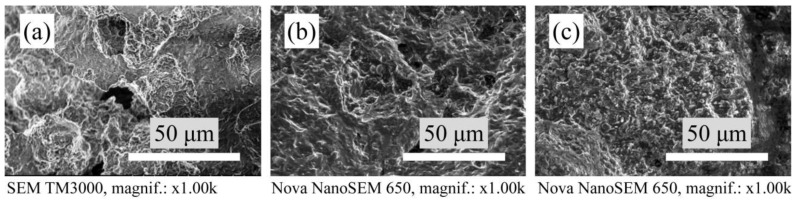
SEM micrographs of the microstructure of sintered samples: (**a**) undoped samples, (**b**) with YSZ and (**c**) with MSZ.

The microstructure of the porous mullite ceramic without a modifying additive and only with yttria-stabilized zirconia or magnesia-stabilized zirconia formed from densely packed and closely bordered crystals is shown in Figure 4.

**Figure 5:**
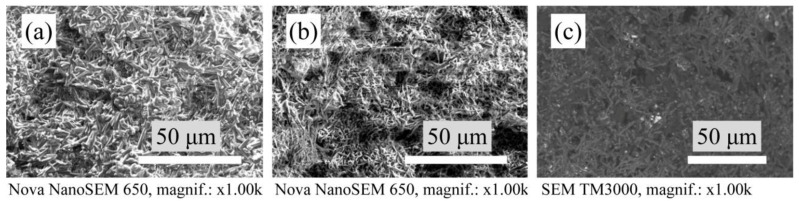
SEM micrographs of the microstructure of sintered samples: (**a**) with YSZ:WO_3_ (1:1), (**b**) with MSZ:WO_3_ (1:1) and (**c**) with YSZ:WO_3_ (1:2).

**Figure 6:**
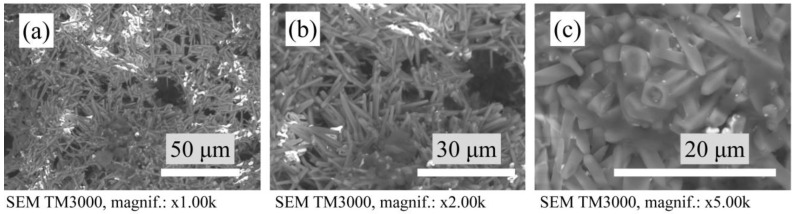
SEM micrographs of the microstructure of sintered samples with MSZ:WO_3_ (1:2): (**a**) magnification ×1.00k, (**b**) magnification ×2.00k and (**c**) magnification ×5.00k.

The use of the ZrO_2_ and WO_3_ mixtures in a 1:1 and 1:2 ratio for modifying porous mullite ceramics caused the formation of elongated needle-shaped mullite crystals that were randomly located in relation to each other (Figure 5 and Figure 6). Mullite crystals slightly border each other, therefore the structure of the samples is not dense.

The samples with a mixture of YSZ:WO_3_ and MSZ:WO_3_ in a 1:2 ratio have relatively thinner mullite crystals with a distinct acicular or needle-shaped mullite crystals–crystals with a narrow thin ending. The samples with a mixture of MSZ:WO_3_ in a 1:2 ratio differ from other sintered materials in that they contain the porous mullite crystals. On the SEM micrographs (Figure 6c), such porous mullite crystals are displayed as elongated needle-shaped crystals with a round hole at the and. It can be assumed that such mullite crystals have internal hollow or voids along the length of the crystals. The formation of hollow mullite crystals occurred due to the formation of the Al_2_(WO_4_)_3_ at 1075–1100 °C [48,49] and the presence of the Al–Si–O agglomerations on the surface of the Al_2_(WO_4_)_3_ particles. This coincides with the results of the investigations of Liu et al. [49]. The mullitization of such Al–Si–O agglomerations occurred at about 1200 °C, with a simultaneous decomposition of the aluminum tungstate from within. Thus, the porous crystals consisted of a mullite shell and a hollow inner part [49].

### 3.4. Porosity and Pore Size Distributions

**Figure 7:**
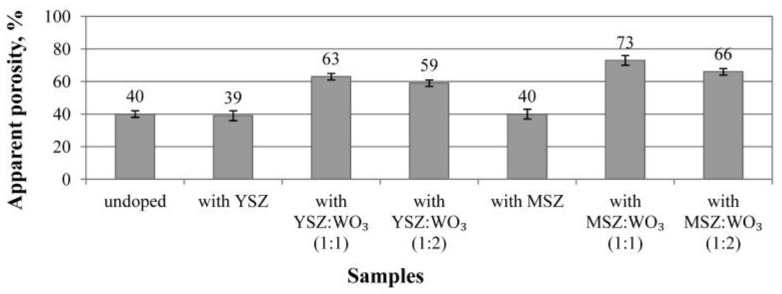
Apparent porosity of the investigated samples.

The apparent porosity of the sintered samples is shown in Figure 7. The samples without a modifying additive and samples with a different stabilized zirconia have a similar apparent porosity of about 40 ± 2%. The samples with mixtures of modifying additives have an apparent porosity higher than 59 ± 2%. 

The use of WO_3_ in the equivalent ratio to zirconia noticeably increases the apparent porosity of the samples in comparison with the undoped samples. Porous mullite ceramics modified with magnesia-stabilized zirconia and WO_3_ in a 1:1 ratio have the highest apparent porosity (73 ± 2%). The doubling of WO_3_ in the MSZ:WO_3_ mixture slightly decreases the porosity (66 ± 2%) of such samples in comparison with the samples with MSZ and WO_3_ in a 1:1 ratio.

**Figure 8:**
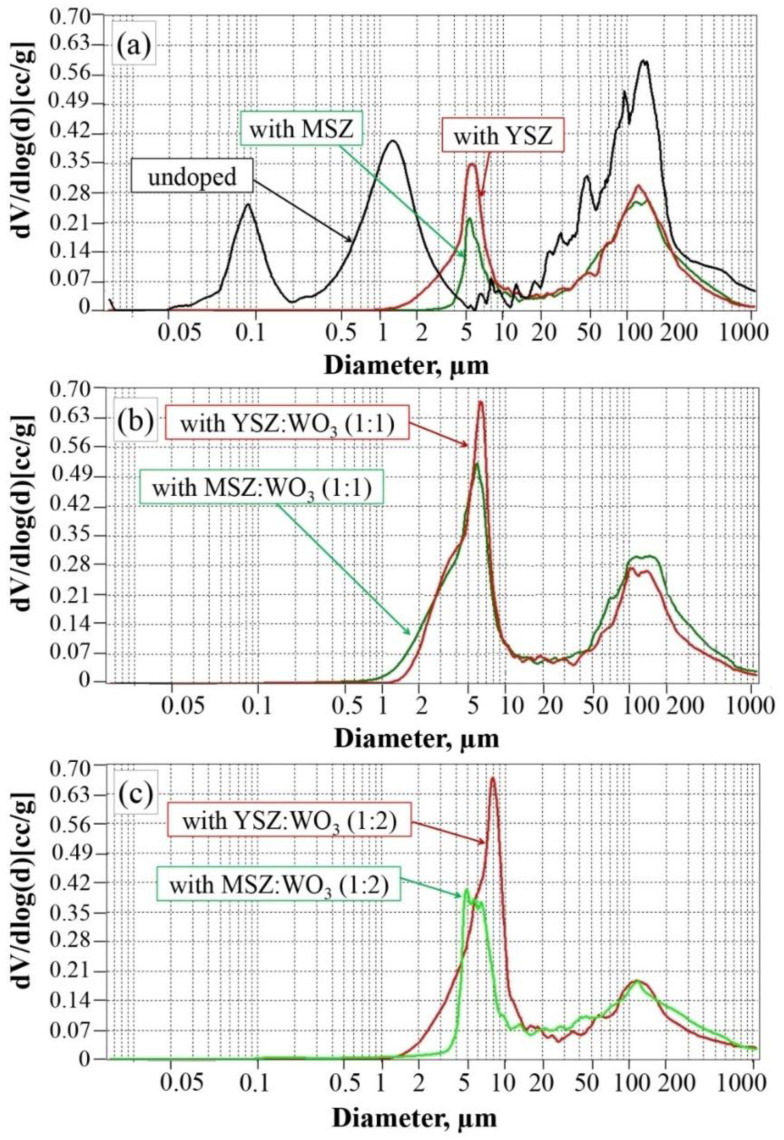
Pore size distributions of the samples: (**a**) undoped samples, with YSZ and with MSZ; (**b**) samples with YSZ:WO_3_ (1:1) and with MSZ:WO_3_ (1:1); and (**c**) samples with YSZ:WO_3_ (1:2) and with MSZ:WO_3_ (1:2).

The graphs of the pore size distribution after the mercury porosimetry are shown in Figure 8. The undoped samples have three ranges of pore size distributions: 0.05–0.2 μm, 0.3–5 μm and 7–1000 μm, with the most pronounced size of ≈0.08 μm, ≈0.15 μm and ≈150 μm in these ranges (Figure 8a).

Two ranges of pore size distributions are observed for the samples with yttria-stabilized zirconia and magnesia-stabilized zirconia (Figure 8a). The biggest pores of these samples are in range from 20 μm to 500 μm, in which ≈150 μm pores are more intensely expressed. Such large pores occupy approximately the same percentage for both compositions. The smaller pores of the samples with the YSZ additive are in the range from 1 μm to 20 μm. For the samples with MSZ, the range of the smaller pores narrows and occupies from approximately 3 μm to 20 μm, and such pores occupy a smaller percentage than in the case of the samples with YSZ. The 5 μm pores are more strongly expressed in the smaller pore size range for the samples of both compositions.

Additionally, the two pore size distribution ranges are in the samples with the YSZ:WO_3_ and MSZ:WO_3_ mixture in a 1:1 ratio (Figure 8b). The range of the smaller pores expanded with the addition of WO_3_, which takes them from 1 to 20 μm, with a pronounced predominance of a 6–7 μm pore size. It can be seen from the graphs in Figure 8b that pores of a smaller diameter (1–20 μm) predominate over the larger sized pores (20–500 μm). The smaller pores of the samples with YSZ:WO_3_ (1:1) occupy a slightly bigger percentage as for the samples with MSZ:WO_3_ (1:1). The 150 μm pores are more intense in the large size pores range samples with an additional oxide mixture in a 1:1 ratio.

The pore size distributions of the samples with the YSZ:WO_3_ and MSZ:WO_3_ mixture in a 1:2 ratio are shown in Figure 8c. The doubling of WO_3_ decreases the formation of large pores. The range of the large pores takes from 20 μm to 1000 μm. The doubling of WO_3_ caused the formation of small pores. The samples with YSZ:WO_3_ (1:2) have pores from ≈1.5 μm to 20 μm, with primarily 6 μm pores. The pores from 4 μm to 10 μm occupy a significant volume of the samples with MSZ:WO_3_ (1:2). The 3–7 μm pores predominate in the case of the samples with MSZ:WO_3_ (1:2).

Pores larger than 1000 μm (or 1 mm) are not shown on the pore distribution graphs, although they are shown in the SEM pictures in Figure 3.

### 3.5. Specific Heat Capacity 

**Figure 9:**
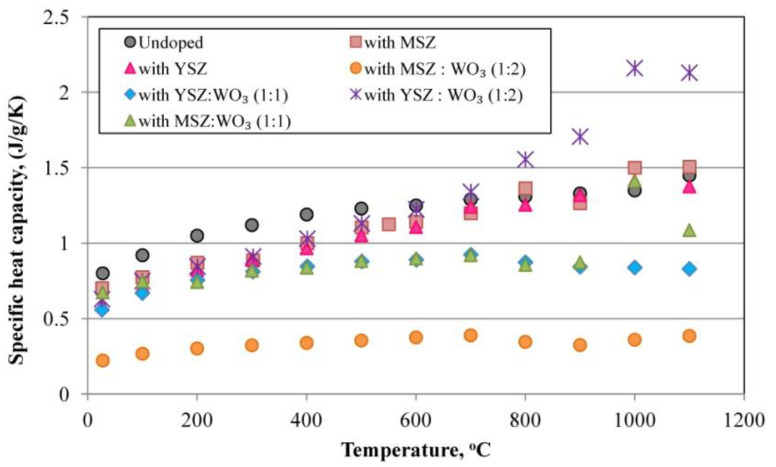
Specific heat capacity of the samples.

Figure 9 shows the temperature dependence of the specific heat capacity of the investigated samples. The approximately 1200–1300 °C specific heat capacity curves of the mullite single crystals and polycrystalline mullite show an anomalous sigmoidal increase [50], which also corresponds to the specific heat capacity lines obtained for the undoped samples and samples modified with YSZ and MSZ, as well as for YSZ:WO_3_ in a 1:1 ratio and MSZ:WO_3_ in a 1:2 ratio. These samples have a similar temperature dependence of the specific heat capacity from 25 °C to 1100 °C and an anomalous sigmoidal increase. The specific heat capacity values of the samples with a mixture of YSZ:WO_3_ in a 1:2 ratio and a mixture of MSZ:WO_3_ in a 1:1 ratio rapidly increase at about 1000 °C and then decrease due to the presence of WO_3_ with a characteristic temperature dependence of the specific heat capacity [51,52,53,54,55]. This can be explained by the fact that the phase compositions of these samples have the pronounced presence of the WO_3_ phase and its phase transitions during heating. Han et al. determined that the WO_3_ phase transitions tetragonal(t_1_) → tetragonal(t_2_) at 1200 K (927 °C) occur with a large increase in the specific heat capacity and with a subsequent decrease [52,56,57,58,59]. In these works, such an increase in the specific heat capacity of the porous mullite ceramics occurs closer to 1000 °C perhaps due to the presence of other phases that can affect the phase transition temperature.

The samples with a mixture of MSZ:WO_3_ in a 1:2 ratio have the lowest specific heat capacity, which does not exceed ≈0.40 J/g/K at all temperature ranges due to the presence of zircon with a relatively low specific heat capacity.

### 3.6. Thermal Conductivity

**Figure 10:**
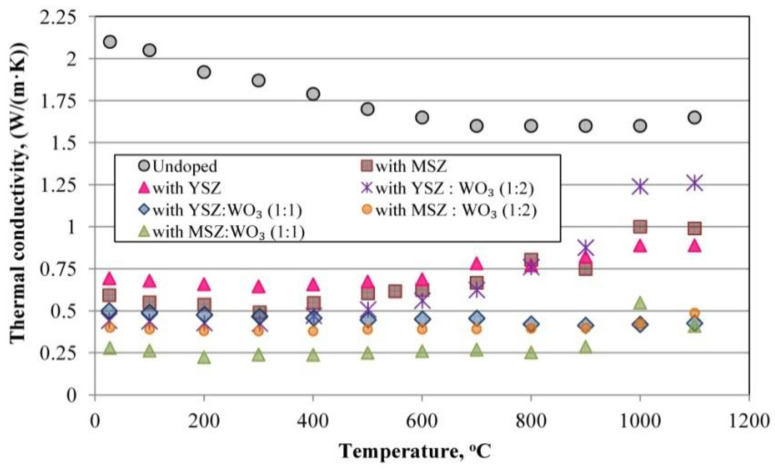
Thermal conductivity of the samples.

Figure 10 shows the thermal conductivity of the sintered porous mullite ceramic samples after the laser flash contactless method measurements. From the point of view of thermal conduction, the porous ceramics can be regarded as a two-phase system [53]. The first phase is the ceramic material skeleton or matrix. The second phase varies as the porosity, pore size and form, as well as the roughness of the pore walls. The heat transfer through such phases forms and describes the common thermal conductivity of the porous ceramic material. The mullite materials are the matrices of the investigated porous ceramics. 

The thermal conductivity of the dense fully mullite ceramic is 5.1 W/mK and the thermal conductivity of the air is 0.026 W/mK [53]. The thermal conductivity of the porous mullite ceramics with a porosity of about 75%, according to the published data, is 0.31–0.42 W/mK at room temperature [53]. The thermal conductivity is directly proportional to the porosity of the investigated samples. Samples with a lower porosity of about 39–40% have the highest thermal conductivity, and porous ceramic samples with a porosity of about 63–73% have a lower thermal conductivity.

The undoped samples with a 40% porosity have a higher thermal conductivity in the temperature range of 25–1100 °C compared to all the modified samples. The thermal conductivity of the undoped mullite porous ceramics with a porosity of ≈40% is 2.1 W/mK at room temperature. The undoped samples also have a high thermal conductivity due to the predominance of isolated, large-sized spherical pores, as well as due to the presence of a corundum phase with a high λ_T=25°C_ = 41.9 W/mK [53,55] that increases the thermal conductivity of the ceramic matrix. The change in the slope on the temperature dependence of the thermal conductivity at temperatures greater than about 1000 °C is due to a radiative contribution to the measured thermal conductivity.

The thermal conductivity of the samples modified with YSZ and MSZ and with a porosity of ≈40% at room temperature is 0.69 W/mK and 0.59 W/mK, respectively. The thermal conductivity of such samples with these compositions increased to 0.82 and 0.74 W/mK at 900 °C. Such parameters as the porosity, microstructure and presence of mullite and monoclinic ZrO_2_ in the samples only with YSZ or with MSZ are similar, but the thermal conductivity is higher for the samples with YSZ due to the presence of a corundum phase with a high thermal conductivity coefficient. The intensity of the corundum phase of these composition samples is much less than that for the undoped samples, therefore, its influence on the thermal conductivity is less than in the case of the undoped samples.

The thermal conductivity of the samples with a porosity of about 59%, 63% and 66% at room temperature is 0.50 W/mK, 0.40 W/mK and 0.28 W/mK, respectively, for samples with a mixtures of YSZ:WO_3_ in a 1:1 and 1:2 ratio and samples with a mixture of MSZ:WO_3_ in a 1:2 ratio. Its λ at 900 °C became 0.41 W/mK, 0.40 W/mK and 0.88 W/mK. The thermal conductivity of the samples with a mixture of YSZ:WO_3_ in a 1:2 ratio increases with an increasing temperature and became about 0.88 W/mK at 900 °C. The thermal conductivity of the samples with a YSZ:WO_3_ mixture in a 1:1 ratio and with MSZ:WO_3_ in a 1:2 ratio does not change intensively with an increasing temperature and remains within 0.40 ± 0.02 at 900 °C.

The sintered samples with a MSZ:WO_3_ mixture in a 1:1 ratio and with a porosity of 73% have the lowest thermal conductivity of the investigated samples. The thermal conductivity of these samples does not change with an increasing temperature until 900 °C. It is 0.28 W/mK at room temperature and 0.29 W/mK at 900 °C.

Considering the point of view of the so-called mullite ceramic skeleton or matrix, the thermal conductivity is lower for those samples with a looser texture and its structure consists of relatively short, thin, randomly located and loosely packed mullite crystals in the structure. The inclusions of other phase’s crystal grains with a low thermal conductivity reduce the overall thermal conductivity of the porous mullite ceramics. This is clearly noticeable in the case of an undoped sample and in the case of a sample with a mixture of MSZ:WO_3_ in a 1:1 ratio, respectively, for the samples with the highest and lowest thermal conductivity. The dependence of the grain-size and crystal size on the thermal conductivity cannot be ignored. The thermal conductivity is lower for the samples with smaller and thin crystals, respectively, for the samples with a mixture of MSZ:WO_3_ in a 1:1 and 1:2 ratio. This is due to the decrease in the phonon mean free path. As a result, the anharmonic phonon scattering within the grain is dominated and this decreases the thermal conductivity [60].

In its turn, the thermal conductivity is a little higher for the samples with MSZ:WO_3_ in a 1:2 ratio than for the samples with MSZ:WO_3_ in a 1:1 ratio. The thermal conductivity of the samples with MSZ:WO_3_ in a 1:2 ratio is the same at different temperatures, due to the presence of different phases, that prevent the increase in the thermal conductivity with an increasing temperature. The stability of the thermal conductivity of the samples MSZ:WO_3_ (1:2) can also be explained by the chaotic arrangement of mullite crystals, which are weakly adjacent to each other. The porous mullite crystals of such samples also decreases the thermal conductivity.

The Increase in the thermal conductivity of the ceramic samples with only YSZ and MSZ or with YSZ:WO_3_ in a 1:2 ratio with an increasing temperature from 600 °C to 1000 °C could be explained by the expressed content of the monoclinic ZrO_2_. Such an elevation of the thermal conductivity with an increasing temperature is a characteristic property of zirconium ceramics [61]. For samples with YSZ:WO_3_ in a 1:2 ratio, such an increase in the thermal conductivity is also caused by the expressed presence of WO_3_. The specific heat capacity and thermal conductivity of WO_3_ increases with an increasing temperature [52]. Accordingly, it is less pronounced for the samples with a mixture of YSZ:WO_3_ in a 1:1 ratio and MSZ:WO_3_ in a 1:1 and 1:2 ratio due to additional phases such as zircon and aluminum tungstate that reduce this effect.

The change in the slope on the temperature dependence of the thermal conductivity at temperatures greater than about 1000 °C is due to a radiative contribution to the measured thermal conductivity.

### 3.7. Thermal Diffusivity

**Figure 11:**
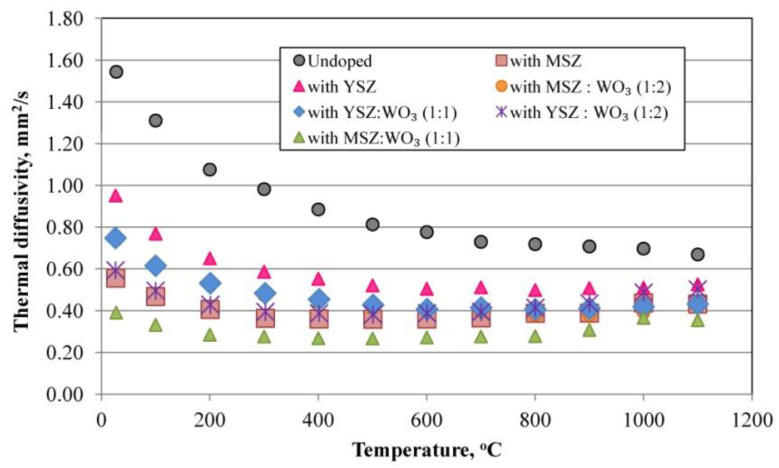
Thermal diffusivity of the samples.

Figure 11 shows the temperature dependence of the thermal diffusivity of the investigated samples. The undoped samples have the larger thermal diffusivity in comparison with the modified porous mullite ceramics, due to a higher thermal conductivity and the similar specific heat capacity in comparison with the modified samples. These undoped samples potentially faster propagate the heat into the medium. 

The modified samples have a relatively similar thermal diffusivity. The samples modified with a mixture of MSZ:WO_3_ in a 1:1 ratio have a lower thermal diffusivity due to the lower thermal conductivity and relatively low specific heat capacity. The low thermal diffusivity of these samples means that heat is mostly absorbed by the material and a small amount of heat is conducted farther.

### 3.8. Thermal Shock Resistance

**Figure 12:**
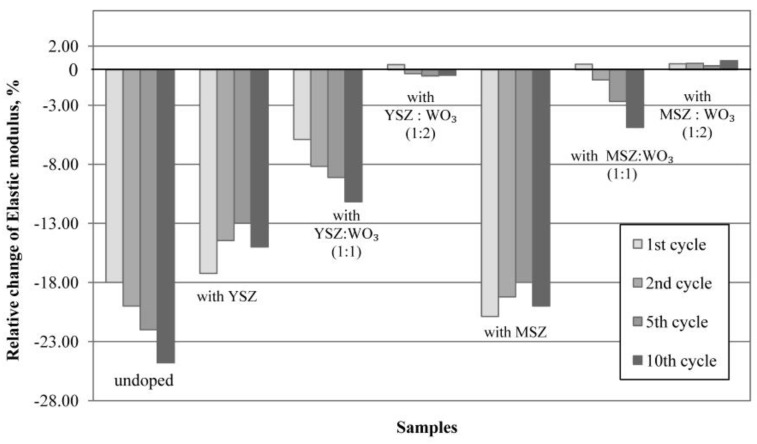
Relative change in the elastic modulus of samples after the thermal shock tests.

The dependence of the relative change in the elastic modulus on the cycle number of the thermal shock is shown in Figure 12. The undoped samples and samples modified with YSZ and MSZ already have a lower resistance to thermal shock after the first cycle of the temperature change corresponding to the scheme 20 °C → 1000 °C → 20 °C with exposure for 1 h at 1000 °C. Such samples lose more than 15% of the elastic modulus after the 10th cycle due to the induced thermal stress after the rapid temperature change. The reading of the elastic modulus of the samples with YSZ and MSZ improved between the 2nd and 5th cycles due to the process of the crack healing of the ceramic materials. Samples with YSZ and MSZ have the ZrO_2_ mainly as a monoclinic (m-ZrO_2_) modification but also as tetragonal (t-ZrO_2_). With a temperature change from 1000 °C to 20 °C, tetragonal ZrO_2_ grains transform into monoclinic grains, thus, the martensitic phase transformation occurs. This phase transformation is accompanied by a volume expansion of 3–4%, which is directed opposite to the crack propagation. The growth of the crack and propagation were prevented due to the induced compressive stress [21,22,61,62,63,64]. However, a further exposure to thermal shock leads to a decrease in the elastic modulus.

The thermal shock resistance of the investigated samples improves with the use of WO_3_ in the additive mixture. In the case of the samples with YSZ:WO_3_ in a 1:1 ratio, the relative change in the elastic modulus is negative already after the first thermal shock test and proportionally reduces with the increase in the thermal shock tests’ numbers in comparison with the undoped samples and samples with YSZ and MSZ. The relative change in the elastic modulus after the 10th thermal shock cycles is ≈11% for the samples with YSZ:WO_3_ in a 1:1 ratio.

The samples doped with a mixture of MSZ:WO_3_ in a 1:1 ratio demonstrate a high resistance to the thermal shock. The elastic modulus of these samples increases by ≈0.5% after the 1st thermal shock cycle and decreases within 5% after the 10th cycle.

The samples with a mixture of YSZ:WO_3_ and MSZ:WO_3_ in a 1:2 ratio have a high thermal shock resistance. The elastic modulus of these samples also increases by ≈0.4% after the 1st thermal shock cycle, but further behavior is different. In its turn, the elastic modulus of the samples with a mixture of YSZ:WO_3_ in a 1:2 ratio decreases after the 2nd cycle and decreases by 0.5% after the 10th cycle. The elastic modulus of the samples with a mixture of MSZ:WO_3_ in a 1:2 ratio does not decrease. The elastic modulus of such samples increases by 0.5% after the 1st thermal shock test and does not decrease after the 2nd and 5th cycle, but it becomes greater than the initial value by ≈0.8% after the 10th cycle.

For samples with a mixture of MSZ:WO_3_ in a 1:1 ratio and a mixture of YSZ:WO_3_ and MSZ:WO_3_ in a 1:2 ratio, the increased thermal shock resistance can be explained by the presence of such crystalline phases as zircon and aluminum tungstate [11,65]. The presence of the aluminum tungstate crystalline phase with a negative linear thermal expansion (α_alumin_. _tungst_. = −1.5 × 10^−6^ °C^−1^) [48] has an influence on the thermal shock resistance of the investigated ceramics. In the case of the aluminum tungstate phase, it shrinks and allows for the expansion of other the crystalline phases of the investigated ceramic without the formation of the internal stresses in the structure at the thermal shock time. Zircon with its low thermal expansion (α_zircon_ = 4.1 × 10^−6^ °C^−1^ from room temperature to 1400 °C) [65] does not increase the expansion of the ceramic samples.

The samples modified with a mixture of MSZ:WO_3_ in a 1:2 ratio together with the thermal insulating ability show the best thermal shock resistance. The polycrystalline structure with chaotic mullite crystals of these samples and a comparable high porosity of ≈63%, as well as the presence of small pores with a pore size range from 4 to 10 μm, do not cause the localization of stresses at the moment of the thermal shock. The relatively small branched pores can deflect, slow down or stop the propagation of cracks by its pinning [66]. The elastic modulus does not decrease as a result of a rapid temperature change due to the compensation of stresses and a reduction in the probability of crack formation and growth. 

## 4. Conclusions

The influence of the porous mullite ceramic modification with different microsized stabilized ZrO_2_ and WO_3_ on the thermal properties was investigated. The porous mullite ceramic with a simultaneously low thermal conductivity and high thermal shock resistance was achieved. The following conclusions were reached:

(a) The use of the microsized ZrO_2_ and WO_3_ additive promotes the formation of elongated partially networked pores with an orientation in a direction parallel to the base of the molds.
(b) The thermal conductivity decreases with an increasing sample porosity and the randomness of the ceramics structure, as well as with the decreasing mullite crystal thickness.
(c) The formation of the hollow mullite crystals decreases the thermal conductivity of the ceramics and stabilizes its temperature dependence.
(d) The increase in the zircon content in the phase compositions of the porous mullite ceramic causes the decrease in the specific heat capacity of these ceramics.
(e) The presence of zircon and aluminum tungstate in the phase compositions of the porous mullite ceramic improves the thermal shock resistance of the investigated ceramics.

Porous mullite ceramics from raw material compositions with a mixture of magnesia-stabilized zirconia and WO_3_ in a 1:2 ratio can be used as a potential thermal-insulating material in conditions of sharp temperature changes.

## Figures and Tables

**Table 1:** Thermal analysis tests.

Thermal Analysis Tests	References
Standard test method for thermal conductivity and thermal diffusivity by modulated temperature differential scanning calorimetry.	[34,35]
Standard test method for determining specific heat capacity by differential scanning calorimetry.	[36,37]
Hot-plate system. Guarded hot-plate systems are used to measure steady-state heat flow through materials with low thermal conductivity (insulators).	[38,39,40,41,42]
Heat flow system. Guarded Comparative–Longitudinal Heat Flow Technique.	[42,43]
The laser flash method and laser pulse: Laser Flash Thermal Conductivity.	[42,44,45]

## Data Availability

The data presented in this study are available on request from the corresponding author.
